# Novel serological neo-epitope markers of extracellular matrix proteins for the detection of portal hypertension

**DOI:** 10.1111/apt.12484

**Published:** 2013-09-15

**Authors:** D J Leeming, M A Karsdal, I Byrjalsen, F Bendtsen, J Trebicka, M J Nielsen, C Christiansen, S Møller, A Krag

**Affiliations:** * Nordic Bioscience, Fibrosis Biology and BiomarkersHerlev, Denmark; † Department of Gastroenterology Faculty of Health Sciences, Hvidovre Hospital, University of CopenhagenCopenhagen, Denmark; ‡ Department of Internal Medicine I, University of BonnBonn, Germany; § Department of Clinical Physiology Faculty of Health Sciences, Hvidovre Hospital, University of CopenhagenCopenhagen, Denmark; ¶ Department of Gastroenterology Odense University Hospital, University of Southern DenmarkOdense, Denmark

## Abstract

**Background**The hepatic venous pressure gradient (HVPG) is an invasive, but important diagnostic and prognostic marker in cirrhosis with portal hypertension (PHT). During cirrhosis, remodelling of fibrotic tissue by matrix metalloproteinases (MMPs) is a permanent process generating small fragments of degraded extracellular matrix (ECM) proteins known as neoepitopes, which are then released into the circulation.

**Aim**To investigate their potential as plasma markers for detection of PHT.

**Methods**Ninety-four patients with alcoholic cirrhosis and 20 liver-healthy controls were included. Clinical and laboratory data of the patients were collected. All patients received HVPG measurement with blood sampling. In these samples, the following degradation or formation markers were measured: C1M (type I-collagen), C3M and PRO-C3 (type III collagen), C4M and P4NP 7S (type IV collagen), C5M (type V collagen), C6M (type VI collagen), BGM (biglycan), ELM (elastin), CRPM (CRP).

**Results**All ECM markers except for CRPM correlated significantly with HVPG. Interestingly, C4M, C5M and ELM levels were significantly higher in patients with HVPG >10 mmHg. Multiple regression analysis identified PRO-C3, C6M and ELM as significant determinants, while the models A and B including PRO-C3, ELM, C6M and model for end-stage liver disease (MELD) provided better description of PHT (*r* = 0.75, *P* < 0.0001). The models provided odds ratios of >100 for having clinical significant PHT.

**Conclusions**These novel non-invasive extracellular matrix markers reflect the degree of liver dysfunction. The different degrees of portal hypertension correlated with these circulating neoepitopes. Using a single blood sample, these neoepitopes in combination with MELD detect the level of portal hypertension.

## Introduction

Development of portal hypertension (PHT) in chronic liver diseases is associated with complications and increased mortality. PHT is defined as a pressure gradient between portal vein and hepatic vein higher than 5 mmHg. The hepatic venous pressure gradient (HVPG) is measured indirectly by catheterisation and wedging a hepatic vein. The formation of varices occurs only at a HVPG above 10 mmHg and a higher HVPG is associated with a poorer prognosis.^1^–^2^ Therefore, the measurement of portal pressure carries important diagnostic and prognostic information and helps to guide the clinical management of these patients. Drawbacks are the invasiveness and availability only in specialised units, rendering HVPG measurement not suitable for screening. Thus, non-invasive assessment of PHT is needed. Even though a number of methods have been evaluated (e.g. combinations of biochemical markers, transient elastography), a simple biomarker – not requiring expensive equipment or trained personnel – would be ideal in any clinical setting.^3^

In advanced stages of fibrosis, the liver contains around six to eight times more extracellular matrix (ECM) proteins than the normal liver.^4^–^5^ ECM mainly consists of types I, III and IV collagen, fibronectin, laminin, hyaluronan, elastin, undulin and proteoglycan.^6^,^7^ During fibrosis, endopetidases such as matrix metalloproteinases (MMPs), especially MMP-2 and MMP-9, are upregulated and involved in the remodelling (degradation and formation) of ECM during the progression of fibrosis.^9^–^12^ The remodelling of ECM proteins leads to MMP-generated peptide fragments acknowledged as neo-epitopes that are released into the circulation. These fragments are uniquely modified and thereby bear specific ‘protein finger-prints’, which are specifically recognised by markers developed by our group for the assessment of the remodelling of structural proteins that are involved in liver fibrosis.^13^ These theoretically driven markers are expected not only to reflect the amount of fibrosis at a given time point but also to reflect hepatic fibrotic activity that may enable the prediction of increase or decrease in fibrosis severity and PHT. This is plausible as these novel markers were designed to assess protein neoepitopes that are generated during fibrosis progression or regression involving up- or downregulation of disease-relevant proteins and proteases. Similar neo-epitopes are clinically and successfully used for these purposes in other ECM-related pathologies such as osteoporosis and arthritis^13^–^14^ These ECM markers show a tight relation to experimental liver fibrosis and PHT as shown previously^15^–^21^; therefore we investigated the ability of these neo-epitopes for the non-invasive assessment of PHT.

## Patients and methods

### Cirrhotic patients

A total of 94 patients with alcoholic cirrhosis admitted to Hvidovre Hospital were included in the study. The aetiology of cirrhosis was alcoholic in 90% of the patients and autoimmunic or post-hepatitic in the rest. Fourteen patients who were referred to a hepatic venous catheterisation to exclude splanchnic ischaemia served as controls. These individuals all had normal liver function and no signs of mesenteric ischaemia. The diagnosis of cirrhosis was based on liver biopsy or by endoscopic proven varices or portal hypertensive gastropathy together with classical clinical, biochemical and ultrasonic signs of cirrhosis. Oesophageal variceal status was assessed during standard routine endoscopy. If the information was available in the patient's medical record, a repeated endoscopy was not performed in relation to this study. The degree of PHT was measured during a liver vein catheterisation in abstinent stable patients without any acute events. Patients with gastrointestinal bleeding within the last 3 weeks before the study, insulin-dependent diabetes, acute or chronic intrinsic renal or cardiovascular disease, alcoholic hepatitis, hepatorenal syndrome, malignant disease, or severe arterial hypertension were excluded. Fasting femoral artery plasma trazylol and hepatic venous plasma trazylol samples were collected in trazylol tubes from patients and stored at −80 °C. Patients participated after giving their informed consent in accordance with the Helsinki II Declaration and the studies were approved by the local ethics Committee for Medical Research in Copenhagen and Danish Data Protection Agency (J-No.2008-41-2020).

### Assessment of hemodynamic parameters and routine biomarkers in patients

Hemodynamic investigations were performed in the morning after an overnight fast and at least 1-h resting in the supine position. Hepatic veins and femoral artery were catheterized. An indwelling polyethylene catheter was placed in the femoral artery and the arterial blood pressures were measured directly by a capacitance transducer. Catheterisation of hepatic veins was performed as previously described.^22^ A Swan-Ganz 7F balloon catheter (Edwards, Irvine, CA, USA) was guided under flouroscopic control to the above locations via the femoral route. Pressures were measured in at least three vessels by a capacitance transducer (Simonsen & Weel, Copenhagen, Denmark) with the midaxillary line being zero pressure level. Mean values of repeated measurements were used. HVPG was determined as the wedged minus free hepatic venous pressures.^23^–^24^ The hepatic blood flow was determined by the indocyanine green constant infusion technique. The indocyanine green clearance (ICG) was measured as the infusion rate divided by the arterial plasma concentration of indocyanine green.^25^ The galactose elimination capacity (GEC) was determined as previously described by Tygstrup.^26^ Bilirubin, albumin and international normalised ratio (INR) were assessed in heparin peripheral plasma samples.

### Stratification of patients

Patients were stratified according to pre-defined levels of PHT^27^: 5 mmHg < HVPG < 10 mmHg = PHT; 10 ≤ HVPG < 16 = Clinically significant PHT with increased risk of decompensation; HVPG ≥ 16 mmHg = Severe PHT with a poor prognosis and high risk of death. Twenty five percent of patients had a PHT below HVPG 10 mmHg.

### Quantification of ECM-related biochemical markers

MMP degraded collagen of types I, III, IV, V and VI (C1M,^15^ C3M,^16^ C4M,^17^ C5M,^28^ C6M,^18^ respectively); MMP degraded CRP (CRPM^29^), MMP degraded biglycan (BGM^30^), MMP degraded elastin (ELM,^31^); formation markers pro-collagen type III (PRO-C3) and the 7S domain of type IV (P4NP 7S^21^), were all assessed in the collected femoral artery plasma and hepatic venous plasma samples. Briefly, each assay was run on a 96-well streptavidin plate coated with the appropriate biotinylated synthetic peptide dissolved in an optimised assay and incubated 30 min at 20 °C. 20 μL of peptide calibrator or sample was added to appropriate wells, followed by 100 μL of conjugated monoclonal antibody raised against the specific sequence of interest and incubated 1 h or overnight at 4 °C or 20 °C. Finally, 100 μL tetramethylbenzinidine (TMB) (Kem-En-Tec cat.438OH) was added and the plate was incubated 15 min at 20 °C. All the above incubation steps included shaking at 300 rpm. After each incubation step, the plate was washed five times in washing buffer (20 mM Tris, 50 mM NaCl, pH 7.2). The TMB reaction was stopped by adding 100 μL of stopping solution (1% H_2_SO_4_) and measured at 450 nm with 650 nm as the reference. A calibration curve was plotted using a four-parametric model. A brief technical summary of the ECM biochemical markers is seen in Table 1. Samples were measured within the detection range. All assays were tested for analyte stability and all were acceptable.

**Table 1:** Overview of technical specifications of the novel ECM assays and CRPM assay presented in this study

Assay name	Target	Antibody type	Detection range (ng/mL)	Intra-assay variation (%)	Inter-assay variation (%)	Ref
C1M	MMP-2/9/13 degraded type I collagen	Monoclonal	0.83–500	10.1	6.7	21
C3M	MMP-9 degraded type III collagen	Monoclonal	0.9–50	4.7	6.5	22
C4M	MMP-2/9 degraded type IV collagen	Monoclonal	0.6–100	4.8	12.1	23
C5M	MMP-2/9 degraded type V collagen	Monoclonal	11.3–1000	4.4	9.1	34
C6M	MMP-2/9 degraded type VI collagen	Monoclonal	0.3–250	4.1	10.1	24
PRO-C3	N-terminal propeptide of type III collagen	Monoclonal	0.9-200	4.1	11.0	38
P4NP 7S	7S domain of type IV collagen	Monoclonal	7.9–500	9.7	11.7	27
CRPM	MMP-1/9 fragment of CRP	Monoclonal	0.8–50	4.2	10.4	35
ELM	MMP-1/12 degraded elastin	Monoclonal	0.48–125	9.4	13.8	37
BGM	MMP-9 degraded biglycan	Monoclonal	3.8–200	5.9	14.9	36

### Statistical analysis

The results of the biomarkers of C1M, C3M, C4M, C5M, C6M, CRPM, BGM, ELM, PRO-C3 and P4NP 7S were logarithmically transformed to obtain normality and symmetry of variance. The demographic characteristics between the groups of Controls and the Child-Turcotte score group were analysed using a one-way Anova with each group as a fixed factor, and the comparison of the distribution of genders was analysed using Fisher's exact test. Comparison of the level of the biomarkers between groups was analysed using a one-way Anova with each group as a fixed factor, and in the pair wise multiple comparisons of each disease group with controls, the level of significance was adjusted using Dunnett's test. Spearman rank-order correlation was used to determine the correlation between single serological biomarkers and HVPG. Multiple linear regression analysis was carried out to assess the relationship between HVPG and a composite marker consisting of a collagen degradation marker, a collagen formation marker and a noncollagen degradation marker. A difference was considered significant if the *P* value was less than 5%. The SAS software package (release 9.2; SAS Institute Inc., Cary, NC, USA) was used for the statistical calculations. The diagnostic potential was calculated as area under the receiver operating characteristic curve (AUROC) using Graphpad Prism 6 (GraphPad Software, Inc., La Jolla, CA, USA) software between healthy controls vs. diseased patients and between patients with and without significant PHT. Prism uses the method of Hanley *et al*.^33^ The standard error is calculated assuming that the area is really 0.5 as the null hypothesis and determines the *P* value from the normal distribution (two-tail). The discriminative potential of the biomarker levels for predicting the level of HVPG was assessed by logistic regression analysis with HVPG stratified into three groups mild (HVPG < 10 mmHg), moderate (10 ≤ HVPG < 16 mmHg) and severe (HVPG ≥ 16 mmHg) PHT; and biomarker levels classified into two groups (≤median, >median). In the logistic regression, the HVPG group was the dependent variable, and biomarker group the predictor variable. Separate models were applied for discrimination of HVPG < 10 mmHg against HVPG ≥ 10 mmHg.

## Results

The demographic description of the patient population is presented in Table 2. Patients with cirrhosis and controls were matched with respect to age and body composition. The distribution of gender was equal throughout the Child-Turcotte classes, while there were more women in the control group. HVPG, model for end-stage liver disease (MELD), INR, and plasma bilirubin increased with increasing Child-Turcotte class significantly. In 26% of patients, serum creatinine was above the normal references range for men (110 μmol/L) and 49% of patients had serum bilirubin above the normal references range (17 μmol/L). The concentration of each marker was tested in arterial vs. hepatic venous blood and was statistically equal (data not shown).

**Table 2:** Demographic data for patients stratified according to the Child-Turcotte classification

	Controls	A	B	C	Anova	Fisher's exact test
*N*	14	32	32	30		
Female/male	8/6	12/20	10/22	5/25		0.05
Age (years)	57.4 ± 12.6	55.0 ± 10.0	55.8 ± 8.9	62.9 ± 14.1	0.16
BMI (kg/m^2^)	24.1 ± 5.1	24.1 ± 4.9	26.3 ± 5.1	23.0 ± 6.5	0.17	
MELD	–	7.4 ± 4.4	13.9 ± 5.0	20.0 ± 4.9	<0.0001
HVPG (mmHg)		7.9 ± 4.6	16.2 ± 4.6	17.8 ± 3.9	<0.0001	
ICG (mL/min)	–	422 ± 173	203 ± 100	110 ± 47	<0.0001
GEC (mmol/min)	–	2.0 ± 0.8	1.4 ± 0.3	1.3 ± 0.3	<0.0001	
Bilirubin(μmol/L)	–	13.1 ± 1.4	20.7 ± 2.7	42.9 ± 4.5	<0.0001
Albumin (mmol/L)	–	595 ± 58	491 ± 80	389 ± 69	<0.0001	
Serum creatinine (μmol/L)	–	73.4 ± 16.5	82.2 ± 34.7	90.1 ± 38.2	0.12

Data are presented as mean ± s.d. Anova test indicates differences for each parameter in the groups.

### Correlation of ECM biochemical markers with liver function

The arterial femoral plasma values of markers correlated almost uniformly with ICG clearance, plasma bilirubin, plasma albumin and Child-Turcotte score (Table 3). In contrast, a weak but significant correlation with GEC, a marker of parenchymatous liver function, was found only for C5M, PRO-C3 and ELM (Table 3). MELD correlated with all liver function and clearance parameters.

**Table 3:** Spearman correlations between ECM markers, CRPM and MELD with single liver function and clinical parameters [HVPG, indocyanine green clearance (ICG), galactose elimination capacity (GEC), bilirubin, albumin, and Child-Turcotte number (Child#)] assessed in arterial femoral plasma

	HVPG	ICG	GEC	Bilirubin	Albumin	Child #
C1M	0.33**	−0.37***	NS	0.21*	−0.30**	0.32**
C3M	0.26*	−0.33**	NS	0.30**	−0.27*	0.34***
C4M	0.36***	−0.42***	NS	0.37***	−0.49***	0.46***
C5M	0.35***	−0.46***	−0.33**	0.35***	−0.30**	0.43***
C6M	0.38***	−0.37***	NS	0.35***	−0.38***	0.42***
PRO-C3	0.47***	−0.55***	−0.31**	0.42***	−0.47***	0.47***
P4NP 7S	0.34***	−0.44***	NS	0.37***	−0.36***	0.37***
BGM	0.36***	−0.34**	NS	0.33**	−0.36***	0.41***
ELM	0.30**	−0.42***	−0.22*	0.25*	−0.28**	0.32**
CRPM	0.19	−0.23*	NS	NS	−0.22*	0.26*
MELD	0.68***	−0.81***	−0.43***	0.86***	−0.67***	0.80***

Data are shown as Spearman's rank correlation coefficients. Asterisks indicate significant correlations each parameter with each marker

* *P* < 0.05;

** *P* < 0.01;

*** *PP* < 0.001).

### Relation of biomarker levels of PHT

**Figure 1:**
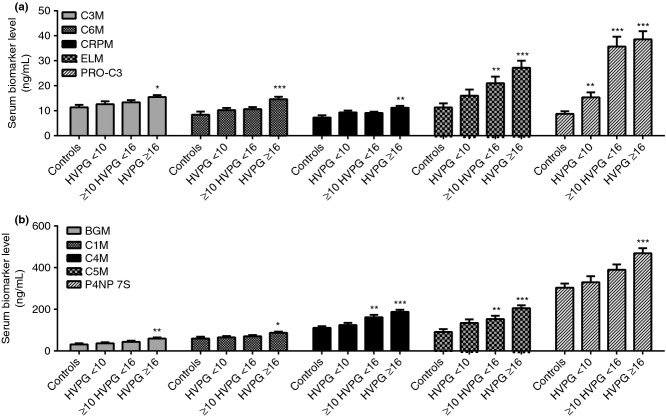
Protein fingerprint markers stratified according to the degree of portal hypertension (HVPG) range: HVPG < 10 mmHg (*n* = 23), HVPG equal 10–16 mmHg (*n* = 28), HVPG ≥16 mmHg (*n *=* *37) compared with controls (*n* = 14). (a) Data for C3M, C6M, CPRM, ELM and P3NP are shown. (b) Data for BGM, C1M, C4M, C5M and P4NP 7S are shown. All data are shown as geometric mean values ± SEM. Asterisks indicate significant difference between the specific groups compared with controls (*P < 0.05; ** *P* < 0.01; ***P < 0.001).

In Figure 1(a) and (b), the plasma levels of the different ECM markers are illustrated in patients stratified according to HVPG level, using the cut-offs of 10 and 16 mmHg. The markers C1M, C3M, C6M, P4NP 7S, CRPM and BGM were significantly elevated in patients with a HVPG above 16 mmHg (*P* < 0.05–0.0001). C4M, C5M and ELM were significantly increased in patients with a PHT above 10 mmHg (*P* < 0.01–0.0001) compared with controls. Lastly, PRO-C3 was elevated in all portal hypertensive patients compared with controls (*P* < 0.01–0001). Table 3 shows the Spearman correlation coefficients between the single biomarker and individual HVPG levels. All plasma biomarkers except CRPM showed a significant and direct correlation with HVPG. Among the collagen degradation markers, C6M exhibited the strongest correlation (*r* = 0.38; *P* < 0.0001). Among the collagen formation markers, PRO-C3 showed the strongest correlation with degree of PHT (*r* = 0.47; *P* < 0.0001), and among the noncollagen markers, the strongest correlation was observed with ELM (*r* = 0.36; *P* < 0.0001). The liver function score MELD exhibited the strongest individual correlation among the parameters analysed (*r* = 0.68; *P* < 0.0001). In a subpopulation of 28 patients (mean HVPG 14.3 ± 6.3 mmHg, range 1.5–24 mmHg), the platelet count was correlated with HVPG providing a significant correlation (*r* = 0.5; *P* < 0.01).

### Multiple marker models for improved detection of PHT

**Figure 2:**
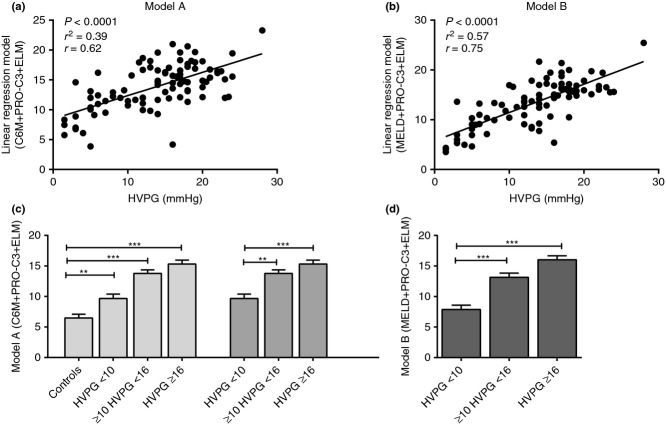
Combination of plasma biomarkers in a linear regression algorithm correlated with HVPG or stratified according to clinical relevant HVPG range in cirrhotic patients and controls. (a) Model A combining ECM markers only correlated with HVPG. (b) Model B combining ECM markers and the MELD score to HVPG. (c) Model A and (d) Model B stratified according to portal hypertension ranges.

**Figure 3:**
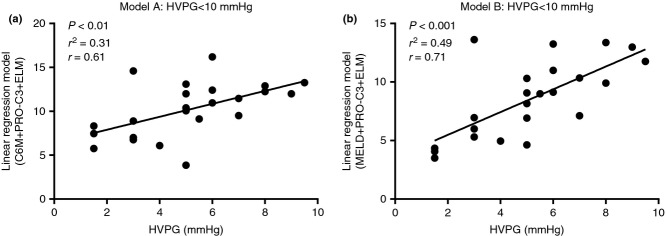
Models A and B correlated with HVPG in patients with a HVPG<10 mmHg (*n* = 23) (a and b, respectively) in cirrhotic patients.

The three strongest biochemical markers were combined in a multiple linear regression model to investigate the potential of a composite model of biomarkers to detect the degree of PHT. The model included C6M, collagen formation markers, i.e. PRO-C3, and noncollagen degradation markers, i.e. ELM. The model including these three markers resulted in model A (Figure 2a: −12.0 + (2.7 × log C6M) + (3.9 × log(PRO-C3)) + (2.0 × log(ELM)) (*r* = 0.62; *P* < 0.0001). The model was further improved by adding the MELD score (model B). After adding the MELD score, the determinant C6M became nonsignificant, and the resulting model B was then defined by: −5.6 + (0.4 × MELD) + (2.8 × log(PRO-C3)) + (1.3 × log(ELM)) (*r* = 0.75; *r*^2^ = 0.57; *P* < 0.0001) (Figure 3b).

Models A and B were tested in patients stratified according to HVPG ranges (Figure 2c, d). Generally, a very low variation was observed in each group and significant differences were observed due to this low variation. Model A values were significantly elevated in all levels of PHT compared with controls (*P* < 0.01–0.0001). Model A and B values were significantly elevated in patients with HVPG ≥ 10 mmHg compared with patients with HVPG < 10 mmHg (*P* < 0.0001). The correlation between model A or B and HVPG was significant in patients with mild PHT (HVPG < 10 mmHg) (Figure 3; Model A: *r* = 0.61 and model B: *r* = 0.71).

### Detection of liver cirrhosis and early PHT

**Figure 4:**
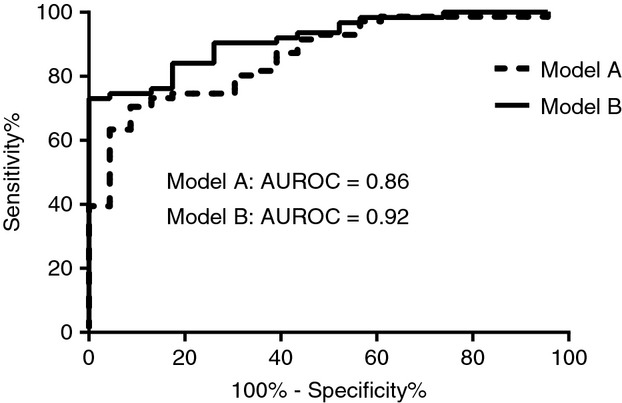
Area under the receiver operating curve (AUROC) plots for models A and B for the separation of HVPG≤10 mmHg vs. HVPG>10 mmHg.

All single ECM markers, CRPM and Model A were able to diagnose cirrhotic patients compared with healthy controls (Table 4); AUROCs were between 0.67 and 0.94 (*P* < 0.05–<0.0001). Most importantly, the majority of the markers (except for C1M, C3M, CRPM) were able to distinguish between mild PHT (HVPG < 10 mmHg) and clinically significant PHT (HVPG > 10 mmHg). The highest diagnostic power among the single markers was observed for PRO-C3 (AUROC = 0.87, *P* < 0.0001) and C4M (AUROC = 0.75, *P* < 0.001). The diagnostic power of PRO-C3 was comparable to the MELD score. Model B provided the highest diagnostic power (AUROC = 0.92, *P* < 0.0001). In Figure 4, the ROC is seen for models A and B displaying the sensitivity and specificity at various cut-off values. In Table 5, the odds ratios of having different levels of PHT are shown. It is seen that patients above the median of MELD, model A or model B are at a significantly higher risk of having mild or moderate PHT compared with moderate or severe PHT (Odds ratio 6.8 to above 100, *P* > 0.01–0.0001). However, model A was not able to separate moderate from severe PHT.

**Table 4:** Diagnostic power of the protein fingerprint markers for the separation of patients with cirrhosis compared with controls or cirrhotic patients with a HVPG less or equal to 10 mmHg compared with patients with a HVPG above 10 mmHg

	AUROC	S.E.	*P* value
C1M			
HVPG ≤ 10 mmHg vs. HVPG > 10 mmHg	0.63	0.07	0.06
Patients vs. healthy controls	0.67	0.08	<0.05
C3M			
HVPG ≤ 10 mmHg vs. HVPG > 10 mmHg	0.63	0.08	0.06
Patients vs. healthy controls	0.69	0.04	<0.05
C4M			
HVPG ≤ 10 mmHg vs. HVPG > 10 mmHg	0.75	0.06	<0.001
Patients vs. healthy controls	0.79	0.05	<0.001
C5M			
HVPG ≤ 10 mmHg vs. HVPG > 10 mmHg	0.67	0.07	<0.05
Patients vs. healthy controls	0.82	0.05	<0.001
C6M			
HVPG ≤ 10 mmHg vs. HVPG > 10 mmHg	0.66	0.07	<0.05
Patients vs. healthy controls	0.71	0.08	<0.05
PRO-C3			
HVPG ≤ 10 mmHg vs. HVPG > 10 mmHg	0.87	0.05	<0.0001
Patients vs. healthy controls	0.93	0.03	<0.0001
P4NP 7S			
HVPG ≤ 10 mmHg vs. HVPG > 10 mmHg	0.70	0.07	<0.01
Patients vs. healthy controls	0.75	0.06	<0.01
ELM			
HVPG ≤ 10 mmHg vs. HVPG > 10 mmHg	0.69	0.07	<0.01
Patients vs. healthy controls	0.80	0.05	<0.001
CRPM			
HVPG ≤ 10 mmHg vs. HVPG > 10 mmHg	0.55	0.07	0.45
Patients vs. healthy controls	0.71	0.09	<0.05
BGM			
HVPG ≤ 10 mmHg vs. HVPG > 10 mmHg	0.66	0.07	<0.05
Patients vs. healthy controls	0.68	0.07	<0.05
MELD (bilirubin_creatinine_INR)			
HVPG ≤ 10 mmHg vs. HVPG > 10 mmHg	0.87	0.04	<0.0001
Model A (C6M+PRO-C3+ELM)			
HVPG ≤ 10 mmHg vs. HVPG > 10 mmHg	0.86	0.04	<0.0001
Patients vs. healthy controls	0.94	0.02	<0.0001
Model B (PRO-C3+ELM+MELD)			
HVPG ≤ 10 mmHg vs. HVPG > 10 mmHg	0.92	0.03	<0.0001

AUROC, area under the receiver operating characteristic curve. Data are shown as the AUROC, a probability of correct diagnosis by each marker or model. The *P* value indicates significance of the AUROC diagnosis compared with the null hypothesis which is an area of 0.5.

**Table 5:** Showing the odds ratios from the discriminant analysis

Odds ratios	Moderate	Severe
MELD		
Mild	6.8**	63.0***
Moderate	–	9.3***
Model A		
Mild	11.3***	26.3***
Moderate	–	2.3^ns^
Model B		
Mild	>100***	>100***
Moderate	–	29.7***

Having a biomarker above median was associated with increased risk of having mild (HVPG < 10 mmHg) or moderate (10 ≤ HVPG < 16 mmHg) PHT vs. severe PHT (HVPG ≤ 16 mmHg).

Asterisks indicate significant correlation of each parameter with each marker (*P < 0.05;

** *P* < 0.01;

*** *P* < 0.001; ns, nonsignificant).

## Discussion

This study evaluated for the first time the ability of these novel ECM neo-epitopes to detect PHT from a simple blood sample in patients with cirrhosis. We demonstrated that these novel ECM markers were highly correlated with the degree of PHT and might detect clinically significant PHT. Furthermore, the combination of three ECM markers in a linear regression model detected PHT better than the markers alone. The detection of PHT could be improved by combining two ECM markers with the MELD score. Finally, the odds ratio of having moderate or severe PHT vs. mild PHT was increased for model A and model B compared with MELD alone.

Different serological ECM-related markers have been described to correlate with HVPG. Among these laminin is the best-studied serological ECM-related marker to date. Laminin levels correlate with PHT; however, these studies include low numbers of patients.^34^–^37^ Serum hyaluronic acid combined with laminin in a linear regression model had a good discrimination capacity to identify PHT (HVPG > 5 mmHg) in 45 patients with liver fibrosis and cirrhosis of different aetiologies (AUROC = 0.82).^38^ Finally, in a prospective study including 130 patients with or without cirrhosis, the ability of FibroTest was evaluated in relation to HVPG.^39^ The AUROC for discrimination of HVPG < 12 mmHg was 0.79, which was not superior to, for instance, platelet count or Child-Turcotte score. Recently, evidence indicated that the von Willebrand Factor Antigen (vWF-Ag) is related to PHT. This was first investigated in 42 cirrhotic patients^40^ and verified in a larger study showing that plasma vWF-Ag was able to predict a HVPG > 10 mmHg.^41^ The study was performed in 285 patients with compensated cirrhosis. The correlation between HVPG and vWF-Ag was *r* = 0.69 and vWF-Ag predicted HVPG > 10 mmHg independently of Child-Turcotte score. Other types of algorithms and biomarkers have been reported to be significantly related to PHT such as the MELD score, serum sodium (s-Na) and MESO index (MELD/s-Na) with poor correlation with HVPG.^42^–^43^

In our study, the single ECM markers correlated with HVPG similarly to what has been previously found for laminin and PIIINP in small studies of cirrhotic patients. PRO-C3 was significantly better related to levels of PHT than the remaining ECM markers and CRPM. Interestingly, these results are in agreement with data from a previous study that showed correlation between PHT and PIIINP assessed in hepatic venous plasma in a low number of cirrhotic patients.^44^ Of note, the PIIINP marker in this study utilised a polyclonal antibody in the assay^44^ providing a poorer correlation with PHT. Our novel PRO-C3 assay employs a monoclonal antibody specific for the C-terminal end of PIIINP, which is released during collagen formation. Therefore, PRO-C3 is a marker of disease activity, which is not the case for former PIIINP assays. In addition, the novel assays of C4M, C5M and ELM correlated with HVPG. Surprisingly, CRPM did not correlate with HVPG in our patients. This might be explained by the reflection of the inflammatory state and acute-on-chronic events with extrahepatic organ dysfunction on these patients, rather than hemodynamic derangements due to PHT. Moreover, it should be kept in mind that these ECM markers assess the remodelling of structural proteins involved in liver fibrosis and that other factors than fibrosis may influence PHT in cirrhosis.^45^ However, a major advantage in applying these markers was related to the combination with a liver function score improving the detection of PHT.

The ability of a single biochemical marker to detect PHT was improved by combination of three markers describing different aspects of fibrogenesis: collagen degradation, collagen formation, and noncollagen degradation. The correlation was significantly improved from *r* = 0.47 using the single markers PRO-C3 to *r* = 0.62 for model A. A multiple marker approach combining the MELD score for liver function and the two novel ECM serological markers for fibrosis, model B, increased the correlation with HVPG to a higher level than observed for MELD alone (*r* = 0.75). It was seen that a single marker such as Pro-C3 performed just as well in relation to the AUC (AUC = 0.87, *P* > 0.001) for the separation of HVPG >10 and <10 mmHg as for MELD, which includes the assessments of three biomarkers. C4M is also seen to perform well as a single marker (AUC = 0.75, *P* > 0.001). The thought is that MELD relates to renal and liver function, whereas the serum ECM markers describe the hepatic ECM remodelling driven by the disease, thus complementing each other. The correlation between each model and HVPG was strong in patients with a HVPG below 10 mmHg, indicating that the markers are able to detect mild PHT. This is in alignment with data found for transient elastography, which assesses the amount of fibrosis at a given time point.^46^–^47^ Model A including ECM marker only showed a higher odds ratio of having moderate PHT compared with mild than MELD alone. The use of model B combining ECM markers with MELD showed that patients above the median had more than a 100-fold risk of having moderate or severe PHT compared with mild PHT. Therefore, the addition of the multimarker approach significantly improves the correlation with HVPG and increases the odds ratio dramatically for the detection of portal pressure level. Needless to say, that this has important clinical implications in the selection of patients for endoscopy and liver vein catheterisations.

As the concentration of each marker was tested in arterial vs. hepatic venous blood and was statistically equal, this indicates that normal venous blood punctures may be used replacing arterial- and hepatic venous blood.

### Limitations

Generally, the analysis of serological biochemical markers using the ELISA technique often has limitations such as background noise from the normal level of tissue remodelling and variation inherited into the method. Here, the signal-to-noise ratio may be improved by incorporation of disease-related post-translational modifications such as protease cleavage into the specificity criteria of a given assay.^48^ The Protein Fingerprint markers reflect fibrotic activity and the majority of the patients had severe fibrosis reflected by a significant elevation of HVPG. The potential of the markers to differentiate between various degrees of liver fibrosis needs to be verified in larger studies including patients with histologically verified fibrosis, but this study is an important step in this direction. In the current study, it was a limitation that it was not possible to analyse patients with low MELD and PHT; this is an aim for further studies.

In perspective, the combination of the novel protein fingerprint fibrosis markers as evaluated in the present study and the routinely used MELD score may provide a new clinical opportunity for improved evaluation of the involvement of hepatic fibrosis activity in PHT in patients with cirrhosis. This may add in the selection of patients for diagnostic procedures and treatments. The novel protein fingerprint serological markers describe the remodelling of structural proteins, which may better portray the activity of hepatic remodelling, which affects metabolic function and PHT. Most likely, the markers are more valuable in patients with early and moderate fibrosis in relation to the estimation of fibrosis progression. However, this needs to be proved in future prospective studies.

In conclusion, the severity of PHT correlates with these novel ECM serological biomarkers. The combination with clinical scores may aid in the detection of the level of PHT using a simple serological sample. In addition, these novel non-invasive ECM markers may reflect the degree of liver dysfunction and could be useful in the selection of patients for endoscopy and invasive pressure measurements. Further studies to investigate the association of these biomarkers and liver pathophysiology and clinical outcome are warranted.

## Authorship

*Guarantor of the article*: Diana J Leeming.

*Author contributions*: DJL: collected the clinical samples and handled the patients. SM, AK, FB: collected biomarker data and analyzed these. DJL, IB, MK, AK: major revision of the manuscript. CC, SM, FB, AK, MK, JT, MJU: evaluation and discussions of data. DJL, JT, MK, FB, SM, AK, IB, MJU, CC: all authors approve the final version of the manuscript.
